# Antibiotic Susceptibility and Clarithromycin Resistance Determinants in *Helicobacter pylori* in the Northeast of Spain: A One-Year Prospective Study

**DOI:** 10.3390/antibiotics12020356

**Published:** 2023-02-08

**Authors:** Saray Mormeneo Bayo, Alba Bellés Bellés, Diego Vázquez Gómez, Montserrat Planella de Rubinat, Diana Carolina Bayas Pastor, Arturo Morales Portillo, Alfredo Jover Sáenz, Éric López González, Núria Prim, Mercè García-González

**Affiliations:** 1 Unidad de Microbiología, Hospital Universitari Arnau de Vilanova de Lleida, Institut Català de la Salut, 25198 Lleida, Spain; 2 Institut de Recerca Biomèdica de Lleida Fundació Dr. Pifarré, IRBLleida, 25198 Lleida, Spain; 3 Servicio de Digestivo, Hospital Universitari Arnau de Vilanova de Lleida, 25198 Lleida, Spain; 4 Servicio de Farmacia, Hospital Universitari Arnau de Vilanova de Lleida, 25198 Lleida, Spain; 5 Unidad Territorial Infección Nosocomial (UTIN), Hospital Universitari Arnau de Vilanova de Lleida, 25198 Lleida, Spain

**Keywords:** *Helicobacter pylori*, clarithromycin resistance, triple therapy

## Abstract

*Helicobacter pylori* is one of the most widespread infections, and it is reaching alarming resistance levels worldwide. The recommended first-line empirical treatment differs according to the local rate of clarithromycin resistance. Macrolide resistance is mainly associated with three point mutations in the 23S rRNA gene. The aim of this study was to describe the antibiotic susceptibility of *H. pylori* in our healthcare area and the main mechanisms involved in clarithromycin resistance. Gastric biopsies (*n* = 641) were collected and cultured in a one-year prospective study. Antibiotic susceptibility testing was performed by gradient diffusion. A multiplex real-time PCR test (Allplex^TM^ *H.pylori* & ClariR Assay, Seegene) was used to detect the most frequent mutations associated with clarithromycin resistance. Overall, 141 isolates were available for antibiotic susceptibility testing. The highest resistance rates were detected in metronidazole and levofloxacin. The rate of clarithromycin resistance was 12.1%, and the associated mutations were A2143G and A2142G. More than half of the clarithromycin-resistant isolates presented high MIC values (>256 mg/L). Tetracycline resistance was not detected, suggesting that therapies that contain tetracycline could be a suitable option. The low clarithromycin resistance rate coupled with the high rates of metronidazole resistance may support the recovery of the classical triple therapy in our healthcare area.

## 1. Introduction

*Helicobacter pylori* colonizes the gastric epithelium and represents one of the most widespread bacterial infections worldwide, infecting approximately half of the world’s population. The spread of *H. pylori* depends on the geographical area, and the prevalence of infection increases with age, poor sanitary conditions and lower socioeconomic status. This infection causes chronic gastritis and peptic ulcer disease. *H. pylori* increases the risk of gastric adenocarcinoma and mucosa-associated lymphoid tissue lymphoma; therefore, it has been designated a class I carcinogen by the World Health Organization [1,2]. *H. pylori* infection has been officially recognized as an infectious disease included in the International Classification of Diseases, leading to the recommendation that all infected patients be treated [3]. Urea breath tests, stool antigen tests and serology are non-invasive techniques that are used to diagnose *H. pylori* infection in uncomplicated patients. Endoscopic evaluation and bacterial culture are required in patients in which empirical treatments or in patients with alarm symptoms such as gastrointestinal bleeding [2].

Empirical treatments include the combination of antibiotics and proton pump inhibitor (PPI) or bismuth. Clarithromycin triple therapy with amoxicillin, clarithromycin and PPI has been the most frequently used first-line treatment. Clarithromycin is a key antibiotic in the treatment of *H. pylori* infection. However, treatment failure with this triple therapy has been reported in about 20% of patients, mainly due to clarithromycin resistance [4]. For these reason, new therapy alternatives are being tested to improve *H. pylori* eradication [5,6]. Non-bismuth quadruple therapy with PPI, amoxicillin, clarithromycin and metronidazole has been recently implemented as first-line treatment. This combination has improved the efficacy of triple therapies in eradication of *H. pylori*. However, the effectiveness of quadruple therapy may be compromised when metronidazole and/or clarithromycin resistance is present. Consequently, patients would be exposed to at least one ineffective antibiotic that would only increase the environmental antibiotic pressure [3,4].

The use and abuse of unnecessary antibiotic treatments are the main cause of the worldwide increase in antibiotic resistance. Resistance rates to previously effective antibiotic treatments in *H. pylori* are of great concern in public health. This alarming scenario requires careful selection of antibiotic prescriptions and a thorough revision of therapeutic strategies. In this context, different aspects related to the clinical role of *H. pylori* infection were re-evaluated in the recent Maastricht VI Consensus Report [3]. Empirical treatments for *H. pylori* eradication should be based on clarithromycin resistance rates. When individual susceptibility testing is not available, the recommended first-line empirical treatment differs according to the local rate of resistance to this antibiotic. Therefore, bismuth quadruple (bismuth, tetracycline, metronidazole and PPI) or clarithromycin triple treatments are the first-line options in areas with clarithromycin resistance rates below 15%. However, clarithromycin triple therapy should not be used when the rate of resistance to this antibiotic is over 15% or unknown. In these situations, the recommendation is either bismuth or non-bismuth quadruple therapies [2].

Nowadays, most patients are empirically treated. As gastric biopsy culture is invasive and expensive, this technique is mainly performed following treatment failure. Accordingly, antibiotic susceptibility testing is not routinely performed in most laboratories [7]. However, clarithromycin resistance rates should be studied in different regions of the world according to the aforementioned recommendations.

Knowledge of the main mechanisms involved in macrolide resistance is essential. Clarithromycin inhibits protein synthesis by binding to the 50S ribosomal subunit in 23S rRNA [8]. Resistance to clarithromycin is mainly associated with three point mutations (A2142G, A2143G and A2142C) in the peptidyl-transferase loop of the 23S rRNA gene [7,9]. Novel technologies to detect mutations associated with clarithromycin resistance include real-time polymerase chain reaction (PCR) or in situ hybridization. These molecular techniques are excellent options when culture is not possible [7,10].

The aim of this study was to describe the antibiotic resistance profile of *H. pylori* isolates from gastric biopsies in our geographical area. The main mechanisms involved in clarithromycin resistance were also evaluated.

## 2. Results

### 2.1. Clinical and Epidemiological Data

A total of 641 gastric biopsies were cultured, and *H. pylori* was isolated in 148 of these specimens by microbiological culture during the period of study. The positivity rate of culture was 23.1%. H. pylori was detected by PCR in another ten samples with negative culture. Therefore, the overall positive rate was 24.6% (158/641). The median age of patients was 56 years (range, 13–88), and 52.5% of them were women.

### 2.2. Antibiotic Susceptibility of H. pylori Isolates

A total of 141 out of 148 isolates were available for AST. The resistance phenotypes of the isolates are shown in Table 1. Most *H. pylori* isolates (85/141, 60.3%) were susceptible to all tested antibiotics. Metronidazole and levofloxacin resistance rates were 17% (24/141). Rates of resistance to clarithromycin, rifampicin and amoxicillin were 12.1% (17/141), 4.3% (6/141) and 3.5% (5/141), respectively. More than half of clarithromycin-resistant isolates presented high MIC values (>256 mg/L). None of the isolates was resistant to tetracycline.

Coresistance to two antibiotics was detected in 8.5 % (12/141) of *H. pylori* isolates. The most frequent coresistance phenotype was levofloxacin plus clarithromycin (*n* = 5), followed by metronidazole and rifampicin (*n* = 2). Four isolates (4/141; 2.8%) were concomitantly resistant to three antibiotics, and two isolates presented with triple amoxicillin, levofloxacin and metronidazole resistance.

Dual clarithromycin and metronidazole resistance was found in one isolate (0.7%), and another isolate presented triple resistance involving clarithromycin, metronidazole and levofloxacin (0.7%).

### 2.3. Molecular Characterization of Clarithromycin Resistance Determinants

A multiplex real-time PCR assay that detects the most frequent mutations associated with clarithromycin resistance in *H. pylori* was performed on 84 biopsies. Determinants of clarithromycin resistance were detected in 19 samples. The most frequently detected mutation was A2143G (15/19; 78.9%), followed by A2142G (4/19; 21.1%). No A2142C mutation was detected.

Culture-based AST was performed in 17 of the samples in which clarithromycin resistance determinants were detected (Table 1), in 12 of which an A2143G mutation was detected, whereas 7 isolates (7/12, 58.3%) presented a clarithromycin MIC value > 256 mg/L, and the remaining had MIC values between 1 and 64 mg/L. Seven isolates with an A2143G mutation were only resistant to clarithromycin. Five were resistant to more than one antibiotic, and the resistance levofloxacin–clarithromycin phenotype was the most frequent. The four isolates with an A2142G mutation showed clarithromycin MIC values > 256 mg/L, none of which presented only resistance to clarithromycin. One clarithromycin-resistant isolate with an MIC value of 16 mg/L did not present any of the tested mutations. The phenotypic susceptibility to clarithromycin correlated with the negative molecular detection of mutations in the 42 tested clarithromycin-susceptible isolates.

### 2.4. Agreement between Pathological Anatomy (PA) and Culture

PA results were available in 623 of 641 cultured biopsies. Results were coincident in 93.4% (582/623) of the specimens processed by PA and microbiology; a total of 122 biopsies were positive, and 460 were negative according to both culture and PA. *H. pylori* was isolated from 22 PA-negative biopsies (22/623; 3.5%) (κ = 0.85).

Nineteen PA-positive samples presented negative culture (19/623; 3%). PCR was performed in these samples, and *H. pylori* was detected in ten of them; the other nine had both negative culture and a negative molecular result. A mutation responsible for clarithromycin resistance (A2143G) was detected in one of these positive samples.

### 2.5. Empirical Antibiotic Treatment

Empirical antibiotic treatment was recorded in 83 patients, 42 of whom (42/83; 50.6%) were treated with non-bismuth quadruple therapy (metronidazole, amoxicillin, clarithromycin and PPI). A combination of bismuth, metronidazole and tetracycline (Pylera^®^) with PPI was prescribed in thirty-four of them (34/83; 41%). The remaining patients (<10%) were treated with other therapies.

## 3. Discussion

Emergence of antibiotic resistance has been one of the main challenges associated with the successful eradication of *H. pylori* worldwide. Given the variation in antibiotic resistance rates in different regions and countries, consensus guidelines recommend that first-line treatment for *H. pylori* be based on primary resistance rates in each geographical area [3]. The main recommendation is to perform clarithromycin AST prior to prescribing the standard first-line clarithromycin-based triple therapy when the resistance rate of this antibiotic is unknown. Therefore, performing AST to determine the clarithromycin resistance rates is essential [11]. Accordingly, the aim of the present study was to determine the antibiotic resistance rates of *H. pylori* in our geographical area and to describe the main determinants of clarithromycin resistance.

Conventional AST requires the isolation of *H. pylori* from stomach biopsies. However, *H. pylori* is a fastidious organism, and culture is challenging and time-consuming. Several factors may hamper bacterial isolation, including the use of PPIs prior to endoscopy or previous antibiotic intake [12]. In addition, biopsies require special transport conditions and should be processed for culture as soon as possible to increase the probability of recovering the isolate. Therefore, a fluid communication between the team performing endoscopies and the microbiology laboratory is essential to maintain a strict transport protocol and to ensure culture success. In the present study, all specimens were processed within the first four hours after endoscopy, which may explain the large number of isolates obtained. In our study, cultures were coincident with PA in most cases and allowed for the performance of AST in most isolates. There was a good overall agreement between microbiology and PA, both regarding positive and negative results.

Antimicrobial resistance patterns of *H. pylori* were previously unknown in our area. The present work shows lower rates in primary antimicrobial resistance than those reported in other areas of Spain or Europe [13,14,15]. In contrast, our local rates were similar to those reported on a study from the northwestern area of Spain [16]. Differences in the geographical area and in the use of antibiotics may contribute to such variation in antimicrobial resistance rates [13,14,15,16,17].

Clarithromycin is essential in combined treatment against *H. pylori*. Macrolides have been widely prescribed for respiratory and genital infections, which has been associated with increasing resistance to this family of antibiotics. A recent systematic review and meta-analysis reported global clarithromycin resistance rates in *H. pylori* of more than 15% [15]. However, the rate of clarithromycin resistance in the present study was 12%. Our results are similar to the frequencies reported in a recent study performed in the north of Spain [16]. In contrast, a high rate of clarithromycin resistance (22.4%) was reported in the aforementioned study performed in a northwestern region of Spain between 2014 and 2016 [13]. Geographical and temporal variation in clarithromycin resistance rates within the same country support the need to perform *H. pylori* AST in different areas and over time. Given the low resistance rate reported herein, the classical triple therapy that includes clarithromycin, amoxicillin and PPI might be considered in our area. In addition, the high rate of metronidazole resistance detected in the present study (17%) supports the use of this triple therapy, in agreement with other European studies [18]. Further clinical studies should be performed to confirm these findings.

Most clarithromycin-resistant *H. pylori* isolates had high MIC values (≥64 mg/L). Clarithromycin resistance in this bacterium is mainly acquired though point mutations in the peptidyl-transferase-encoding region of the V domain of the 23S rRNA gene. Five different point mutations have been reported to be associated with macrolide resistance in *H. pylori* strains: G2115A, G2141A, A2142C, A2142G and A2143G [19,20]. The latter two were the only mutations detected in our specimens. The predominance of these two single mutations in our study is in agreement with other reports on *H. pylori* [21,22]. The A2143G mutation has been previously associated with different levels of resistance [21,23]. The same authors reported the association of mutations at position 2142 with a high level of macrolide resistance. Accordingly, mutants with A2143G showed a broad range of clarithromycin MIC (1–256 mg/L), whereas the four strains with an A2142G mutation had MIC > 256 mg/L in our study. Given that *H. pylori* contains two copies of the 23S ribosomal DNA gene, a higher macrolide MIC may be obtained when the two of them are mutated. Mutations in either one or the two copies may have contributed to the differences in the range of clarithromycin MIC values observed in our study, as previously described [23].

Other less common mutations have also been associated with clarithromycin resistance. A2142C point mutation has been less frequently reported in previous studies, and it was not detected in our case [24,25]. Additionally, the three mutations that were analyzed in the present study were not detected in one clarithromycin-resistant isolate (MIC = 16 mg/L). This result suggests the involvement of either less common mutations [26] or different mechanisms of resistance encoded outside of the 23S rRNA such as an increase in efflux pump expression, which affects not only macrolides but other antibiotic families as well [27]. This fact underlines that commercially PCR-based kits focused on 2142 and 2143 locus detection might lead to false-negative interpretations [28]. Therefore, these commercial assays should be carefully used, and negative results should be interpreted according to clarithromycin AST.

Antibiotic resistance in *H. pylori* is increasing worldwide and is associated with the overuse of antibiotics [29]. The antibiotics with the highest resistance rates in our study were metronidazole and levofloxacin (both above 15%). These high resistance rates may contribute to a reduction in the eradicating effectiveness of regimens that include these two antibiotics [30]. Metronidazole resistance is the most frequently reported resistance in *H. pylori* worldwide [15]. Nevertheless, many treatment schemes contain metronidazole, which increases their impact in the gastrointestinal microbiota.

Levofloxacin is commonly used for *H. pylori* eradication as a second-line treatment. Fluoroquinolones have been extensively prescribed for respiratory and urinary tract infections [31], a fact that may have influenced the high levofloxacin resistance rate detected in our *H. pylori* isolates. However, our data show that metronidazole and levofloxacin resistance rates were lower than in previous reports [13,14,15,18,22]. The rate of fluoroquinolone consumption is high worldwide, not only in human medicine but also in animal health and agriculture sectors. Decreasing the use of fluoroquinolones has become a priority in One Health approaches due to the ecological impact of these antibiotics and recent safety concerns [32,33]. A primary care antimicrobial stewardship program (ASP) has been developed in our health area. Its implementation has led to a significant reduction in fluoroquinolones prescription and resistance rates over the past 5 years [34]. This ASP might have favored the reduced resistance rates of levofloxacin in comparison with other reports. Therefore, active surveillance of resistant *H. pylori* isolates should be continued.

*H. pylori* resistance to amoxicillin, tetracycline and rifampicin is not frequent [31,35]. Resistance to amoxicillin and rifampicin in our isolates was low (<5%), in accordance with the reports of other authors [15,35]. These results differ from those reported in Asian regions and in other countries such as the United States of America [31,36]. Regarding tetracycline, a range of resistance rates in *H. pylori* up to 14% was reported in the aforementioned meta-analysis [15]. In the present study, all *H. pylori* isolates were susceptible to this antibiotic, which supports the use of tetracycline in bismuth-containing quadruple therapies in our area, in agreement with other reports [18,35].

Our study is subject to some limitations. Given that culture-based AST was only available in patients undergoing endoscopy, the real prevalence of *H. pylori* could not be determined. Therefore, our antibiotic resistance results may be biased, and resistance rates reported here cannot be generalized in other areas. As a strength, the prospective design of this study and the inclusion of an interdisciplinary team allowed for the quick performance of culture. The high rate of successful cultures was correlated with PA results. Additionally, this was the first study conducted in our area to determine the antibiotic resistance rates in *H. pylori* and the main determinants involved in clarithromycin resistance. In fact, only a few reports in our country address this issue, with a low number of isolates included compared to our study.

A worldwide lack of local data may result in empirical prescriptions with broader-spectrum therapies than probably required. Knowledge of the local antibiotic susceptibility patters of *H. pylori* is essential to choose the most appropriate empirical treatments.

## 4. Materials and Methods

A prospective study was performed from October 2021 to December 2022 at Hospital Universitari Arnau de Vilanova (Lleida, Spain), a referral tertiary hospital covering an area of the northeast of Spain with approximately 340,000 inhabitants. A total of 641 gastric biopsies from patients who underwent routine gastric endoscopy were collected and immediately transported to the laboratory of microbiology for *H. pylori* culture and antibiotic susceptibility testing (AST). Patients’ medical records were checked to collect demographic data (sex and age) and information about empirical treatment prescriptions. All available results from pathological anatomy (PA) were analyzed. The overall agreement between microbiology and PA results was evaluated and measured using Cohens’ kappa coefficient (κ).

Biopsies were introduced into a sterile tube with 0.5 mL saline and processed within four hours after sample collection. Prior to inoculation, biopsies were homogenized using a glass mortar in a small volume of saline, and then they were cultured on a commercial selective medium for *H. pylori* (PYLO agar, bioMérieux) and Schaedler agar (bioMérieux). Plates were incubated in microaerophilia at 37 °C and checked for growth after a 3-day incubation. Plates were discarded as negative after a ten-day incubation. Bacterial identification was performed by matrix-assisted laser desorption/ionization time-of-flight mass spectrometry (MALDI-TOF MS, Bruker Daltonics). Six antibiotics (amoxicillin, levofloxacin, clarithromycin, tetracycline, metronidazole and rifampicin) were included in the AST, which was performed by gradient diffusion using Etest strips (bioMérieux) on Mueller–Hinton agar supplemented with 5% horse blood. Categorization of the antibiotic susceptibility results was performed using the following susceptibility breakpoints according to European Committee on Antimicrobial Susceptibility Testing (EUCAST) criteria [37]: amoxicillin ≤ 0.125 mg/L, levofloxacin ≤ 1 mg/L, clarithromycin ≤ 0.25 mg/L, tetracycline ≤ 1 mg/L, metronidazole ≤ 8 mg/L and rifampicin ≤ 1 mg/L.

A multiplex real-time PCR assay (Allplex ^TM^ *H.pylori* & ClariR Assay, Seegene) that detects *H. pylori* and the most frequent mutations (A2142G, A2143G and A2142C) associated with clarithromycin resistance was performed on 84 biopsies according to the manufacturer’s instructions [10]. Genomic DNA was purified from 200 µL of each sample and eluted in 60 µL with the Virus mini kit v2.0 in an EZ1 system^®^ (Qiagen GmbH). The PCR was analyzed by CFX96^®^ (Bio-Rad Laboratories, Hercules), and results were obtained using Seegene Viewer software. These specimens corresponded to clarithromycin-resistant *H. pylori* (*n* = 17), positive cultures but non-viable isolates for AST (*n* = 6) and negative cultures with a positive result from pathological anatomy (*n* = 19). The first 42 clarithromycin-susceptible strains were also included to evaluate the performance of this molecular technique.

## 5. Conclusions

In conclusion, the quick performance of culture allowed for considerable recovery of *H. pylori* isolates, providing knowledge on their antibiotic susceptibility. The lack of resistance to tetracycline in our isolates suggests that the use of this antibiotic in bismuth-containing quadruple therapies as a suitable option. The clarithromycin resistance rate below 15% and the high rates of metronidazole resistance may support the use of triple therapy with clarithromycin, amoxicillin and PPI in our area. As antibiotic susceptibility patterns depend on each geographical area and may vary over time, empirical antibiotic treatments should be based on local information.

## Figures and Tables

**Table 1:** Antibiotic resistance phenotypes and clarithromycin resistance determinants in *H. pylori*.

*H. pylori* Isolates (*n* = 141)	Resistance Phenotype^R^	Clarithromycin Resistance Determinants (*n* Isolates)
85	-	-
16	MET^R^	-
13	LEV^R^	-
8	CLA^R^	A2143G (*n* = 7) *
5	LEV^R^, CLA^R^	A2142G (*n* = 2) A2143G (*n* = 3)
3	RIF^R^	-
2	MET^R^, RIF^R^	-
2	AMX^R^, LEV^R^, MET^R^	-
1	AMX^R^, CLA^R^	A2142G
1	AMX^R^, MET^R^	-
1	CLA^R^, MET^R^	A2143G
1	LEV^R^, MET^R^	-
1	LEV^R^, RIF^R^	-
1	AMX^R^, LEV^R^, CLA^R^	A2142G
1	LEV^R^, CLA^R^, MET^R^	A2143G

AMX: amoxicillin; CLA: clarithromycin; LEV: levofloxacin; MET: metronidazole; RIF: rifampicin. ^R^Resistance phenotype. * No clarithromycin resistance determinants were detected in one CLA^R^ isolate.

## Data Availability

Not applicable.
